# Supplementary material for: Causal interplay between lactose intolerance and gut microbiota: a combined bidirectional Mendelian randomization and in vivo validation study
Source: Front Nutr. 2026 Jun 1;13:1803337. doi: 10.3389/fnut.2026.1803337 (PMC13265576; doi:10.3389/fnut.2026.1803337)
Supplement: Supplementary file 1 [file Data_Sheet_1.zip › supplementary materials/Forward/forest plot/ebi-a-GCST90027689.finngen_R12_E4_LACTONAS.pdf]

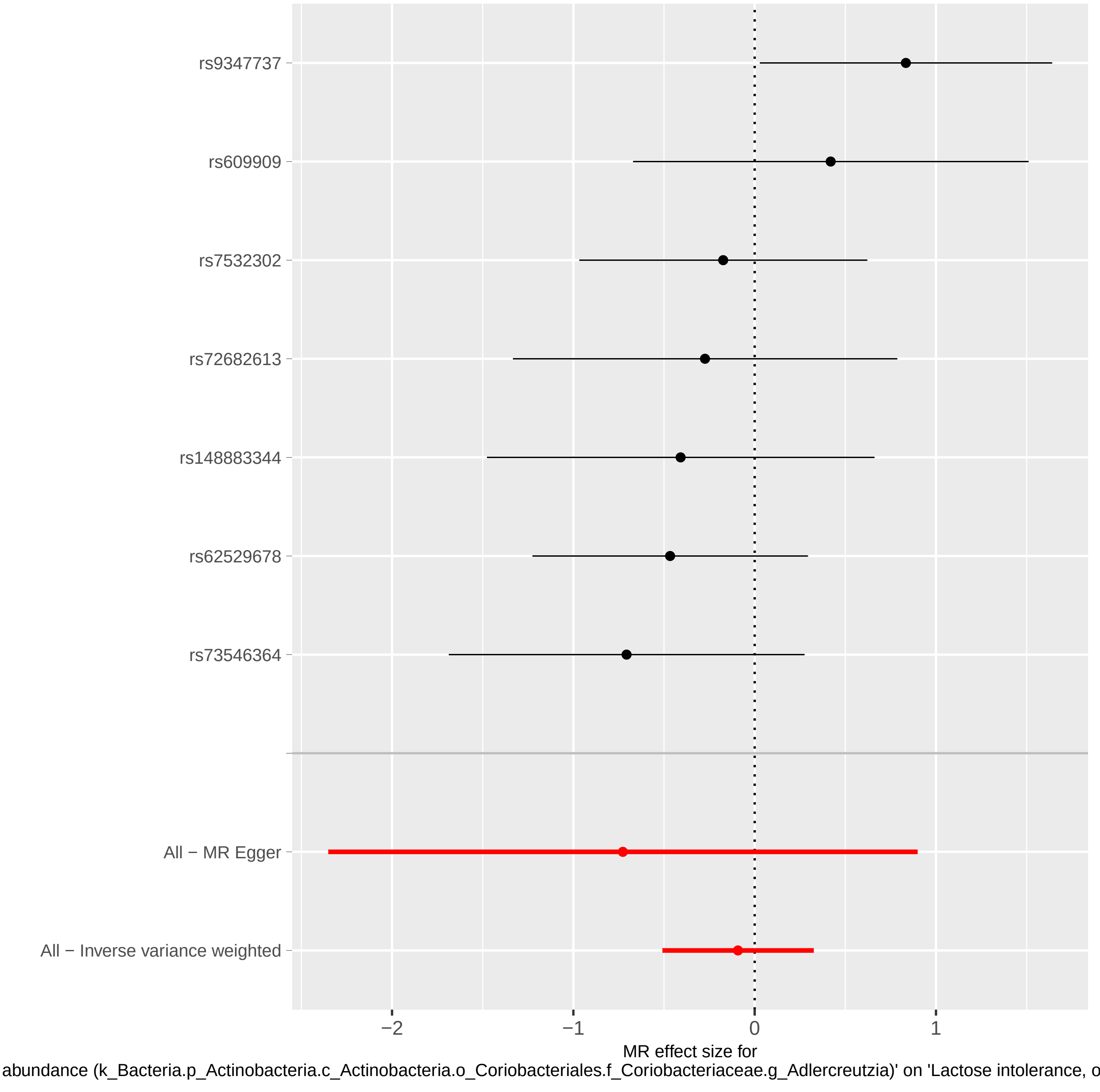
rs9347737
rs609909
rs7532302
rs72682613
rs148883344
rs62529678
rs73546364
All − MR Egger
All − Inverse variance weighted
−2
−1
0
1
MR effect size for
abundance (k_Bacteria.p_Actinobacteria.c_Actinobacteria.o_Coriobacteriales.f_Coriobacteriaceae.g_Adlercreutzia)' on 'Lactose intolerance, o
